# Transplantation of Ovary

**Published:** 1914-01

**Authors:** 


TRANSPLANTATION OF OVARY. Dr. Hugh H. Davidson of London, Eng., reports three cases in the *Medical Review* of St. Louis, Aug., 1913. The first was unsuccessful. In the second, a uterine fibroid, not previously palpable, developed after the operation and the author very candidly questions whether it might not have been due to the ovarian stimulus. The third case was successful but the first menstruation, 3½ months after operation, was profuse, lasting two weeks, in spite of ergot. The next menstruation lasted 4 days, under ergot, but was painless. Since then she has menstruated every month for nine days.

